# Circular economy approach to eggshell waste utilisation: Insoluble protein extraction and CaCO_3_ upcycling for carbonated hydroxyapatite (cHAP)-based fire-resistant wood

**DOI:** 10.1371/journal.pone.0351943

**Published:** 2026-06-25

**Authors:** Edita Garskaite, Stephen R. Euston, Jozef Martinka, Peter Rantuch, Konrad Wilkens Flecknoe-Brown, Patrick van Hees, Michael Försth, Alexandra Byström, Dietrich Buck, Dick Sandberg

**Affiliations:** 1 Division of Building Materials, Department of Building and Environmental Technology, Faculty of Engineering, LTH, Lund University, Lund, Sweden; 2 Institute of Chemistry, Faculty of Chemistry and Geosciences, Vilnius University, Vilnius, Lithuania; 3 Institute of Biological Chemistry, Biophysics and Bioengineering, School of Engineering and Physical Sciences, Heriot-Watt University, Edinburgh, United Kingdom; 4 Department of Physics, Toronto Metropolitan University, Toronto, Canada; 5 Faculty of Materials Science and Technology in Trnava, Slovak University of Technology in Bratislava, Trnava, Slovakia; 6 Division of Fire Safety Engineering, Department of Building and Environmental Technology, Faculty of Engineering, LTH, Lund University, Lund, Sweden; 7 Division of Structural and Fire Engineering, Department of Civil, Environmental and Natural Resources Engineering, Luleå University of Technology, Luleå, Sweden; 8 SWECO, Luleå, Sweden; 9 Wood Science and Engineering, Department of Engineering Sciences and Mathematics, Luleå University of Technology, Skellefteå, Sweden; 10 Department of Manufacturing and Civil Engineering, Norwegian University of Science and Technology, Gjøvik, Norway; University of Gothenburg: Goteborgs Universitet, SWEDEN

## Abstract

Transitioning to a resource-efficient and sustainable circular economy is vital for tackling climate- and environmental-related challenges. This study demonstrates a closed-loop strategy for upcycling agricultural biowaste eggshells. Water-insoluble proteins were extracted from both shell membranes and shell fragments by boiling in water using protein denaturants. The ground eggshells also were used to prepare calcium acetate (Ca(CH_3_COO)_2_) and to treat Scots pine (*Pinus sylvestris* L.) sapwood. Mineralisation of the wood was achieved by performing a two-step impregnation process using aqueous solutions of ammonium dihydrogen phosphate (NH_4_H_2_PO_4_) and Ca(CH_3_COO)_2_ salts. Morphological studies revealed the relatively low saturation of wood matrix with mineral, with cell lumina mostly unfilled, while elemental mapping confirmed homogeneous distribution of Ca and P within the wood matrix. Powder X-ray diffraction (XRD) analysis revealed that wood treatment resulted in the *in-situ* co-precipitation of low-crystallinity hydroxyapatite (Ca_10_(PO_4_)_6_(OH)_2_), and spectroscopic analysis indicated carbonate substitution within the Ca_10_(PO_4_)_6_(OH)_2_ crystal lattice, suggesting the formation of carbonated hydroxyapatite (Ca_10-x_(PO_4_)_6-x_(CO_3_)_x_(OH)_2-x-2y_(CO_3_)_y_). Microscale combustion calorimeter (MCC) and cone calorimeter (CC) measurements of mineralised wood revealed a reduction in the total heat release (THR) compared with untreated wood, indicating potential for further optimisation of wood modification process. Results suggest that the proposed aqueous solution-based processing approach for converting an abundant resource, chicken eggshells, into value-added products has potential for new technology and bioeconomy development and represents a promising pathway towards improved sustainability.

## 1 Introduction

Effective agricultural waste management is essential for environmental sustainability and economic viability [1–3]. Chicken eggshells, classified as animal by-products under EU legislation, are designated as hazardous waste and require costly disposal [4,5]. Mismanagement of this waste leads to environmental hazards, including malodour, microbial proliferation, contamination of soil and water, as well as the release of greenhouse gases into the atmosphere. According to future market insights (FMI), the global egg and egg products market was valued at USD 15.1 billion in 2025 and is projected to reach USD 32.6 billion by 2036 [6]. Based on FMI forecasts, eggshell waste, which constitutes approximately 11% of an egg’s weight, represents a growing environmental challenge [4,7]. Thus, developing strategies to upcycle eggshell biowaste into valuable materials is crucial for advancing resource-efficient and sustainable economies [8,9].

Chicken eggshell is a highly structured composite material having both inorganic and organic fractions [10,11]. The inorganic phase constitutes 95% of the shell, while the remaining 5% consists of organic matrix, including eggshell membrane, and water [11]. Organic matter in the eggshell matrix and the eggshell membranes could be a valuable source of proteins, which have high value in themselves [12–14]. These bioactive molecules exhibit interesting characteristics, *e.g.,* moisture retention and biodegradability, and thus have potential use in clinical, cosmetic, nutraceutical and nanotechnology fields [13,15,16]. Fundamentally, the organic phase of eggshell matrix consists of extractable proteins, which have been studied extensively and many of them have been identified, and insoluble (non-extractable) proteins, which have been studied less [12–14]. Literature shows that extraction and analysis of insoluble eggshell matrix proteins is still a critical technical challenge for biotechnology, as it is laborious and time-consuming. From the technological side, extraction procedures must balance purity, desired yield, cost-effectiveness and environmental impact, [13,16,17] and complexity of the eggshell matrix, formation of insoluble protein aggregates, and protein chemical stability are factors influencing the extractability and analysis of these proteins.

With respect to the inorganic phase, eggshell is a compelling source of calcium, as the calcified zone consists of about 94% of calcium carbonate (CaCO_3_) in the form of mineral calcite [11,18]. Calcite can serve as a precursor for calcium phosphates (CaPs), a group of materials used in the field of bone tissue engineering, as they are not harmful or toxic to the living body [19,20]. Hydroxyapatite (Ca_10_(PO_4_)_6_(OH)_2_, HAP) along with calcium-deficient hydroxyapatite (CDHA), tricalcium phosphate (TCP), and biphasic calcium phosphate (BCP), are the most frequently investigated synthetic CaPs [20,21]. Such minerals, due to their high hardness and compressive strength, high melting points, and chemical inertness, are excellent candidates to be considered as reinforcement material, *e.g.,* for wood protection against fire.

Improving wood reaction to fire is a critical challenge for the modern construction sector. The growing adoption of wood in buildings is driven by its favourable properties, environmental benefits, and advancements in mass-timber systems that enable faster, safer, and more cost-effective construction [22]. Studies showed that numerous compounds containing the elements B, P, Al, and N are effective fire-retardant materials [23,24]. However, the majority of fire retardants currently used in industry to treat solid wood are water-soluble, meaning that active ingredients may leach out when the treated wood is exposed to moisture [25]. This degradation alters the wood’s fire performance and may compromise occupant safety in the event of a fire. Thus, the need for effective fire-retardant treatments that persist through a product’s service life is undeniably high.

Wood modification offers a solution to prevent leaching of fire-retardant additives. A crosslinking process, *i.e.*, chemically joining wood macromolecules to additive compounds via a covalent bond, is an effective way to ‘fixate’ these additives within the wood structure to enhance its durability. For instance, esterification of wood hydroxyl groups with organophosphorus and organoboron compounds has demonstrated improved fire resistance [26]. Similarly, *in situ* polymerisation of furfuryl alcohol blended with ammonium dihydrogen phosphate (NH_4_H_2_PO_4_) has been shown to enhance fire retardancy in poplar wood [27]. Improved leaching resistance of fire-retardant modified wood was also demonstrated when guanyl-urea phosphate, boric acid (H_3_BO_3_), and melamine formaldehyde were combined [28]. However, regulatory concerns over boron-based additives, including H_3_BO_3_ and borax, which are classified as substances of very high concern (SVHC), highlight the need for safer alternatives [29].

Wood mineralisation is an alternative treatment that could enhance wood thermal stability. Silica (SiO_2_), [30] calcium carbonate (CaCO_3_), [31,32] and struvite (MgNH_4_PO_4_·6H_2_O) [33] have been successfully synthesised within the wood matrix. More recently, Scots pine wood was mineralised with brushite (CaHPO_4_·2H_2_O) using NH_4_H_2_PO_4_ as a precursor, leading to notable changes in its thermal degradation [34]. Nitrogen phosphate salts are widely recognised as effective fire retardants due to their availability, low cost, solubility in water, and minimal toxicity [24]. It is, therefore, advantageous to find solutions that allow the fixation of such chemicals within the biopolymeric framework, to develop wood-based composites with enhanced fire-retardant properties and increased resistance to leaching.

Considering the processing of calcium phosphates, coprecipitation from aqueous solutions is the most common synthesis method. This wet-chemistry approach provides a cost-effective means of producing phase-pure materials with controlled crystallinity and particle size. Additionally, synthesis conditions – including precursor selection, solvent composition, concentrations, temperature, pH, mixing time, and reaction duration – can be precisely adjusted to tailor material properties [21,35]. These processing parameters significantly influence the intercalation of inorganic material within the wood matrix. Thus, controlling crystallinity, phase composition, and particle size is essential to improving stability and reducing mineral leakage from the wood structure.

In this study, we provide conceptual insights for circular and resource-efficient material flows that align with key sustainability goals. It is hypothesised that a relatively simple, scalable and adaptable water-based process can be developed to extract insoluble eggshell matrix proteins. Furthermore, it is proposed that the inorganic fraction of eggshells can serve as a precursor for producing calcium phosphate (CaP) mineral-reinforced wood, thereby enhancing its fire-retardant properties.

## 2 Materials and methods

### 2.1 Extraction of insoluble proteins

Chicken eggs were purchased from a local Tesco supermarket, Edinburgh, UK. Eggs (n = 6) were checked to be crack free, washed by brushing their surface, and soaked in distilled water for 24 h. After removing the yolk and white, the eggshells were washed again with distilled water and then soaked for several hours (6 eggshells in 1 L of distilled water). Internal eggshell membranes were removed manually by mechanical peeling, and individual shell fragments were ground with a pestle in a porcelain mortar. In the following step, 2.33 g of internal membranes and 20.00 g of ground eggshells were soaked in 40 mL and 50 mL of 5% acetic acid solution, respectively, and left for 24 h to decalcify. Acetic acid solutions were prepared from glacial acetic acid (CH_3_COOH, > 99%, Fisher Chemicals, Fisher Scientific UK, Bishop Meadow Road, Loughborough, UK). Solutions were then removed by decanting, and solid residues (membranes and eggshells) were centrifuged. These centrifuged residues were then used further to extract insoluble proteins. 1.71 g of ground eggshells was soaked in 10 mL of a dissociating and reducing buffer (0.5 M TRIS/HCl, 1% SDS, 10 mM DTT) and 2.04 g of membrane was soaked in 10 mL of buffer and boiled for 40 min in closed conical polypropylene Falcon centrifuge tubes (capacity 50 mL) placed in boiling water in a glass flask on a heating plate. After boiling, the solution was removed, centrifuged, and 0.5 mL of the supernatant solution was mixed with 0.5 mL of Laemmli 2x (Sigma, S3401-10VL) buffer. This solution was denatured for 5 min in boiling water, then loaded at 10 µL/well and 20 µL/well for sodium dodecyl sulphate-polyacrylamide gel electrophoresis (SDS-PAGE) (S1 and S2 Figs). Gels were run at constant voltage (180 V). After separation, protein bands were visualised by staining gels using a Pierce Silver Stain Kit (Thermo Fisher, UK).

### 2.2 Processing of CaCO_3_ and preparation of impregnation solutions

The dried at room temperature eggshells for 24 h were ground in a ceramic mortar and then calcined in a muffle furnace at 850 °C for 5h with a heating rate 5 °C/min to obtain calcium oxide (CaO). The obtained CaO powder was further ground in a ceramic mortar and dissolved in an aqueous solution of glacial CH_3_COOH (∼50% by volume). The resulting solution was evaporated and dried in an oven at 100 °C for 48 h. Obtained white crystals of calcium acetate (Ca(CH_3_COO)_2_ were subsequently used to prepare an aqueous solution, in which the Ca^2+^ ion concentration was quantified to be 0.5 M (the synthesis procedure is reported elsewhere [36]). In the following step, an adequate amount of ammonium dihydrogen phosphate (NH_4_H_2_PO_4_, 99% Aldrich) was dissolved in distilled water to prepare 1 L of a 0.3 M NH_4_H_2_PO_4_ solution. The pH of the prepared solution was adjusted to 10.4–10.5 using an aqueous ammonia solution (NH_4_OH, 35%, Sigma-Aldrich). The molar ratio of Ca:P in the initial solutions was 5:3.

### 2.3 Mineralisation of Scots pine wood

Specimens of Scots pine (*Pinus sylvestris* L.) sapwood having dimentions of 10 cm × 10 cm × 1 cm and 1 cm × 1 cm × 1 cm (tangential (T) × radial (R) × longitudinal (L)) were cut from sawn timber obtained from northern Sweden (Skellefteå region). The prepared wood blocks were mineralised in a desiccator using a cyclic, two-step impregnation procedure, which is described in detail elsewhere [36]. Briefly, wood blocks first underwent Cycle I impregnation using a 0.5 M Ca(CH_3_COO)_2_ solution. After the impregnation, wood samples were left to dry at room temperature (~18–20°C) for 24 h. This step allows evaporation of the solvent, which facilitates diffusion of the salt solution into the matrix during a Cycle II impregnation. The dried wood samples were then submerged into a 0.3 M NH_4_H_2_PO_4_ solution and connected to a vacuum pump to remove air from the internal parts of the wood matrix. After the impregnation, the mineralised wood samples were dried at room temperature for 30 days and subsequently used for cone calorimeter (CC) tests. The densities for the mineralised wood and the untreated reference wood were calculated to be 549 ± 40 kg/m^3^ and 548 ± 31 kg/m^3^ (± values represent standard deviation (SD)), respectively. Density values correspond to the samples tested in the CC.

### 2.4 Characterisation

Thermogravimetry (TG) and differential scanning calorimetry (DSC) were performed using a PerkinElmer STA 6000 Simultaneous Thermal Analyzer at the Institute of Chemistry, Vilnius University. Dried samples of ~ 5–10 mg were heated from 25 to 855 °C at a heating rate of 10 °C/min in a N_2_ atmosphere (20 mL/min). Mineral phase composition was evaluated using a PANalytical X’Pert Powder diffractometer (Cu Kα radiation, step 0.02° over a 2-theta range of 5–85° at room temperature, exposure time ~96 s per step), at the School of Engineering and Physical Sciences, Heriot-Watt University. Scanning electron microscopy (SEM) and energy dispersive spectroscopy (EDS) analysis were performed on the FEI Quanta 650 FEG SEM (field emission gun scanning electron microscope) equipped with Aztec software from Oxford Instruments, at the School of Energy, Geoscience, Infrastructure and Society, Heriot-Watt University. To generate images, a backscattered electron (BSE) detector was used, with electron beam energy of 15 kV, spot number of 4.0, and chamber pressure of 0.82 Torr. ImageJ software was used to obtain line intensity profiles of SEM image, which plot the variations in grayscale intensity along a specific line and represent the contrast changes across a feature. The ImageJ software was also used to define a horizontal reference line corresponding to the bottom of the wells on the SDS-PAGE gel, from which the distance travelled by each protein band down the gel was measured. To calculate the retention factor (R_f_) for each protein band, the distance travelled by the band was divided by the distance that the Coomassie blue marker dye travelled down the gel. A standard curve was then constructed for the protein molecular weight (MWt) standard markers that is used to estimate the molecular weight of the unknown protein bands. The standard curve was found to give a better fit to a plot of R_f_ versus log_10_(MWt). The raw data for R_f_ for the protein standards and unknown proteins, and details of the fitting procedure are given in the Excel spreadsheet in the Supplementary Materials. Infrared (IR) spectra of mineralised wood were recorded using a Fourier transform infrared (FTIR) spectrometer (Frontier FT-IR, Perkin Elmer; ZnSe/Diamond ATR crystal, DTGS detector, 4000–600 cm^−1^, 4 scans), at the Wood Science and Engineering, Lund University of Technology. Drawings of the lab dishes used in a simplified processing scheme (Fig 3.) were extracted from SciDraw.io and chemix.org depositories. Images representing wood and eggshells were created by using ChatGPT by uploading the original images taken during the experimental work).

**Fig 1:**
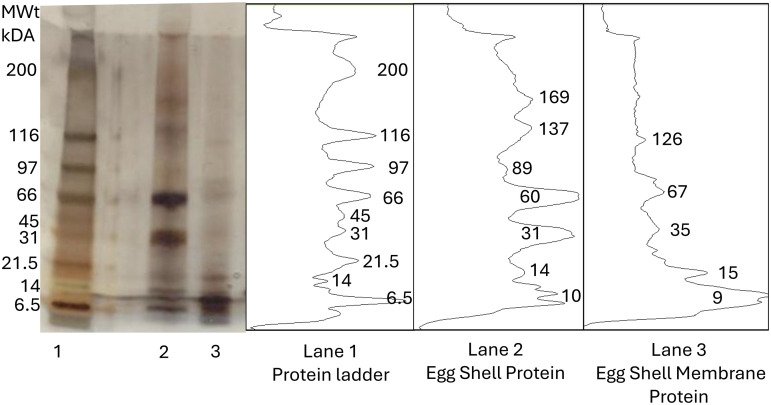
SDS-PAGE electrophoretic profile for insoluble proteins of eggshell matrix and outer membranes. After extraction of proteins by boiling in TRIS/HCl/SDS/DTT buffer solution, the SDS-PAGE gel was stained with silver stain. Lane 1 = molecular marker shows proteins of known molecular weight (MWt) standards (Bio-Rad, broad range) with standard protein molecular weight marked in kDa), lane 2 = extracted protein sample from the eggshells, lane 3 = extracted protein sample from the eggshell membranes. 10 µL/well of sample was used. The contour plots were obtained by defining an equally sized rectangle within each of the three wells and analysing the contour using ImageJ. The migration value R_F_ was determined from the line plot and used to construct a standard curve for the protein standards in lane 1, which was used to calculate molecular weight from the R_F_ value of protein bands in lanes 2 and 3. The molecular weight of each band corresponds to the numbers on the contour plot.

**Fig 2:**
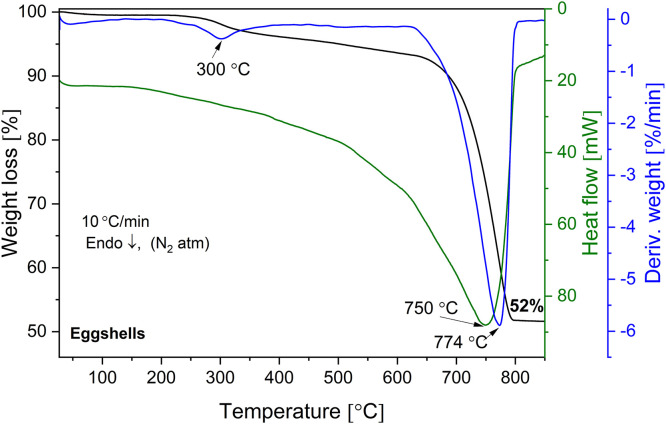
TG/DSC and DTG curves of the eggshells annealed up to 850 °C.

### 2.5 Fire performance test

Before analysis, specimens were conditioned in a climate chamber at 23 ± 2°C and 50 ± 5% relative humidity (RH) for 10 days. Fire performance of samples was investigated by CC (Fire Testing Technology, Ltd., East Grinstead, UK) at two heat fluxes (20 kW m^−2^ and 50 kW m^−2^) at the infrastructure at Faculty of Materials Science and Technology in Trnava, Slovak University of Technology in Bratislava). The CC and testing procedure were in compliance with ISO 5660-1:2015 [37]. The CC measured mass loss of sample, amount of oxygen consumed, and time to ignition (TTI). These data were used to calculate heat release rate (HRR), total heat release (THR) and effective heat of combustion (EHC) in compliance with ISO 5660-1:2015 [37]. From these measured and calculated values, other key fire characteristics were calculated for the investigated samples using the following methods: maximum average rate of heat emission in compliance with Marquis *et al.*, [38] and flashover category in compliance with Kokkala *et al.* [39]. Two replicates for unmodified and mineralised wood samples were tested for each heat flux measurement.

Microscale combustion calorimetry (MCC) testing was performed at Lund University, Sweden, using the apparatus developed by the US Federal Aviation Administration (FAA) [40]. CaP-mineralised wood was studied in oxidative and pyrolysis conditions, *i.e.,* in synthetic air (20% O_2_/80% N_2_ by volume) and N_2_ atmospheres with a heating rate of 1 °C/s. For studies in N_2_ atmosphere, three reference samples of unmodified wood and three samples of CaP-mineralised wood were used; for studies in air, one reference and one CaP-mineralised wood sample were used. CaP-mineralised samples were cut from the internal part of the wood block using a microtome to form stubs 100 μm in thickness. Samples weighed ~5 mg.

Results are presented in heat release rate per unit mass (HRRPUM) for MCC and heat release rate per unit area (HRRPUA) for CC in Sect 3.4.

## 3 Results and discussion

### 3.1 Protein extraction

The SDS-electrophoretic pattern of the eggshell matrix and membrane proteins that were extracted under denaturing conditions is shown in Fig 1. The SDS-PAGE revealed that the precipitate of the eggshell matrix, *i.e.*, of the calcified layer (lane 2), consists of seven distinct migration bands between 10 and 170 kDa. The two broad and strongest lines with molecular masses of 31 and 60 kDa could be assigned to ovocalyxin and ovocleidin proteins. The complex array of bands, including those of ovocalyxin and ovocleidin, in SDS-PAGE of eggshell matrix proteins, has been observed and reported previously [12,41–43]. Ovocleidins and ovocalyxins have been proposed to be important in regulation of eggshell mineralisation and anti-microbial defence [10,44]. Several narrow bands with molecular masses of 20 kDa in SDS-PAGE were also present, including a narrow unidentified band of 10 kDa and a narrow band of 14 kDa. The latter band may be assigned to lysozyme, which is found in both the shell membranes and the calcified shell matrix [41,45–47]. Three bands with molecular weights between 89 and 169 kDa were also present (Fig 1, lane 2) but exhibited reduced staining intensity relative to the bands described above. These bands remain unidentified, but may correspond to ovocleidin-116 [10,42]. Literature reports that the amount of protein in eggshell (mg/g of eggshell) varies, [48] and numerous factors – such as presence of different ions and their concentration, as well as pH, temperature, and extraction duration – affect proteins’ solubility and consequently their detection [49,50]. A different profile was observed for the eggshell membrane (Fig 1, lane 3). The SDS-PAGE showed lower staining intensity, and predominantly low molecular weight proteins were present [51]. A broad band of ~9–11 kDa, consisting of several overlapping bands, may represent ovoglycoprotein, a protein of egg’s white [52]. Another distinct narrow band was observed at 15 kDa and assigned to lysozyme protein [45]. A broad, lower intensity band present at 35 kDa was assigned to the ovocalyxin-36 protein [44,53,54]. Membrane extracts also exhibited bands around 67 and 126 kDa; the former may correspond to ovotransferrin and the latter band remains uncharacterised [47,52]. Gels were also run with double the amount of sample per well (20 µL) (SDS-PAGE gel is presented in S2 Fig). These gels exhibited profiles with more intense bands of the specific proteins already mentioned, as expected, but no additional bands were observed. Further analysis*, e.g.*, dialysis, column chromatography, or mass spectrometry, is needed for accurate sample quantification as well as to confirm protein structure [55].

### 3.2 Eggshell inorganic matrix processing

TG, DSC and derivative thermogravimetric (DTG) curves for eggshells are shown in Fig 2. Three main steps of weight loss were clearly seen in the DTG curve. The first, very small weight loss of ∼0.5% was observed by heating the sample to 100 °C and assigned to the removal of absorbed water. The second step of weight loss (∼2.5%) occurred up to 350 °C (maximum at ∼300 °C, DTG curve). This loss can be attributed to the release of volatile components such as CO, CO_2_, H_2_O, and low-molecular-weight nitrogen-containing compounds, resulting from the decomposition of organic material present in the eggshells. Heating to 850 °C produced an additional ∼44% weight loss (maximum at 774 °C, DTG curve) and a simultaneous exothermic reaction (DSC curve) with a maximum at 750 °C. This loss was assigned to CO_2_ release due to decomposition of CaCO_3_ [56]. The decomposition of the eggshells was completed at approximately 800 °C, and the inorganic residue of about 52% is consistent with the expected CaO yield when CaCO_3_ content of the eggshell is considered. Based on reaction (1), the theoretical mass yield of the CaO from pure CaCO_3_ is approximately 56%:


$$\begin{array}{c} {\text{CaCO}}_{\text{3}}(s)=\text{ CaO }(s)+{\text{CO}}_{\text{2}}(g)\;\;\Delta \text{H }=\text{ 178 kJ}/\text{mol} \end{array}$$ (1)

**Fig 3:**
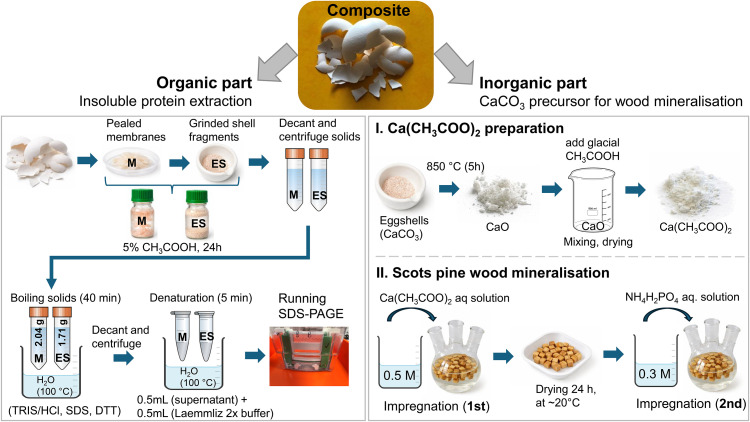
A simplified scheme of the eggshell biowaste processing based on circular economy principles. (M – membranes, ES – eggshells).

A simplified scheme of the eggshell biowaste processing performed in this work is presented in Fig 3. The scheme illustrates the processing pathway of both organic and mineral phases of the eggshells, as well as subsequent wood mineralisation steps.

### 3.3 Calcium phosphate-mineralised wood

**Fig 4:**
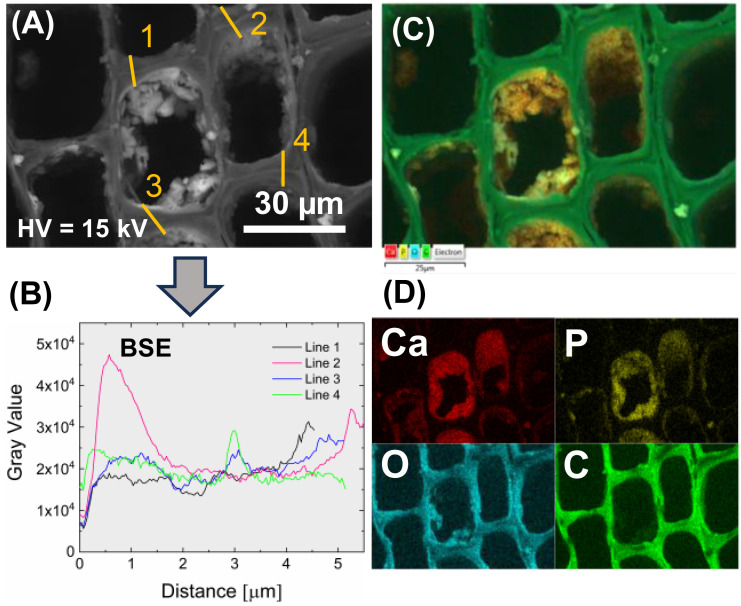
SEM/EDS images of the mineralised Scots pine sapwood. Micrometre stubs cut from the internal section of a 1 cm × 1 cm × 1 cm cube: (A) backscattered electron (BSE) image showing deposited mineral within wood cell lumina and marked four lines across cell walls used to estimate mineral deposition throughout the wood matrix, (B) graph shows plotted horizontal lines (processed with ImageJ) from image **(A)**; (C) and (D) shows distribution of individual elements within wood matrix (colours assigned as Ca – red, P – yellow, O – cyan, C – green).

To assess Scots pine wood matrix saturation and distribution of the CaP mineral within wood cell walls, SEM/EDS analysis was used. Cross-section SEM images, depicted in Fig 4, show morphological features of the inner part of the mineralised wood block. Treatment preserved the open porous structure of the wood matrix, whilst a layer of CaP mineral was deposited on the cell walls. Different areas of the BSE image (Fig 4A) show different brightness intensities, and the light grey areas indicate aggregated particles of co-precipitated mineral. Furthermore, horizontal line analysis across cell walls in the BSE image (Fig 4A and 4B) showed that the middle lamella exhibits an enhanced light grey area that indicates cell wall mineralisation. Distribution of elements within the wood matrix was evaluated through EDS analysis (Fig 4C and 4D). This EDS-based elemental mapping showed homogeneous distribution of Ca and P within wood matrix (Fig 4D) and confirmed the migration of Ca^2+^ and phosphate ($\overset{\text{3}-}{\underset{\text{4}}{\text{PO}}}$) ions through the entire wood block. Saturation of the cell wall with different ionic species and mineral precipitation within the wood matrix has also been demonstrated using vacuum-pressure impregnation in our previous studies [57]. Furthermore, the surface of treated wood blocks exhibited a different morphology, *i.e.,* the amount of mineral precipitated on the surface was larger compared to that observed within the internal layers of wood blocks. The reaction between Ca^2+^ ions and $\overset{\text{3}-}{\underset{\text{4}}{\text{PO}}}$ ions in a system of 0.5 M Ca(CH_3_COO)_2_ and 0.3 M NH_4_H_2_PO_4_ solutions is instantaneous, and accumulation of precipitate on the wood surface during cyclic impregnation is therefore inevitable. Several studies have shown that surface treatment could be an important factor for enhanced protection or additional functionality of wood material [58,59]. Thus, mineralisation of wood matrix with CaP minerals could be a cost-effective method of treatment that also extends the lifetime of wood products.

**Fig 5:**
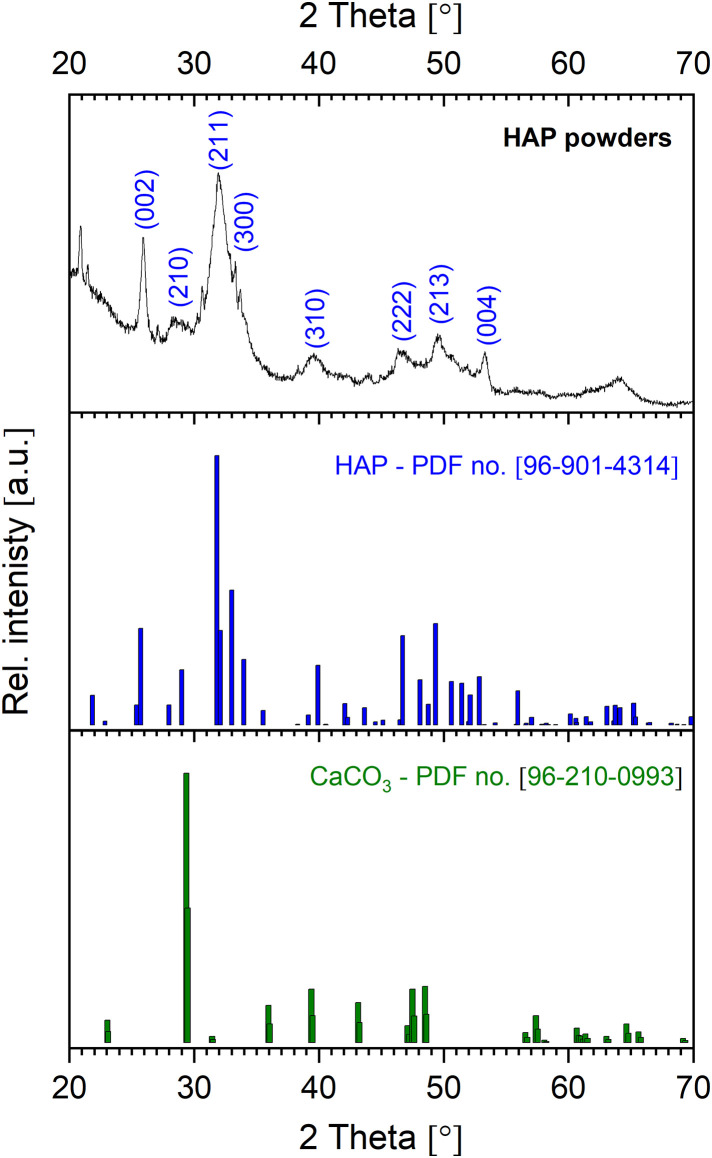
XRD pattern of powders precipitated after the Cycle II wood impregnation treatment. Powders dried at room temperature. XRD pattern showing main Bragg reflections assigned to the polycrystalline HAP phase, and column patterns for the powder diffraction file (PDF) no. 96-901-4314 (for HAP) and no. 96-210-0993 (for CaCO_3_).

To confirm the crystalline phase of the mineral formed within wood matrix, the powders coprecipitated during wood impregnation (cycle II treatment) were removed from an aqueous impregnation (‘parent’) solution, dried at room temperature, and analysed by recording its XRD pattern (Fig 5). Main reflections in the diffraction pattern were observed at 2θ = 25.9°, 28.2°, 31.9°, 33.3°, 39.5°, 46.5°, 49.5°, and 53.3° and assigned, respectively, to the (002), (210), (211), (300), (310), (222), (213), and (004) diffraction peaks of the polycrystalline HAP phase, which is consistent with literature data (JCPDS No. 96-901-4314, hexagonal crystal system, space group P 63/m) [20,60]. The addition of NH_4_OH to NH_4_H_2_PO_4_ solution was a critical step as HAP phase formation is highly pH-dependent. Ca^2+^ ions were combined with $\overset{\text{3}-}{\underset{\text{4}}{\text{PO}}}$ ions at pH 10.4–10.5 to avoid the formation of secondary, less-stable CaP phases. Additional small reflections in the diffraction pattern may arise from the crystallisation of CaCO_3_, calcite (JCPDS No. 96-210-0993, trigonal crystal system, space group R-3c), and from the crystallisation of various ionic compounds formed during solvent evaporation from the co-precipitated mineral, as the powders were not washed following drying. The influence of pH, Ca/P ratio in solutions and processing conditions (stirring or static system) on the polymorphs and crystallinity of the apatitic precipitate and CaCO_3_ has been reported previously [61,62]. Considering that the CaP precipitate is formed within wood material, it is expected that the hierarchical complexity and molecular composition of the matrix affect the diffusion and concentration of ionic species inside the wood cells, and thus the coprecipitation and purity of a formed solid material [63]. XRD analysis also showed that the reflections in the diffraction pattern are broad indicating a small crystallite size. To comprehend the structural characteristics and guide process optimisation, determination of the degree of crystallinity as well as proportion of each phase formed may be required. Additional investigations, such as transmission electron microscopy (TEM) and/or Rietveld refinement analysis to confirm the crystal phases, may also be necessary to further support optimisation of the wood mineralisation process.

**Fig 6:**
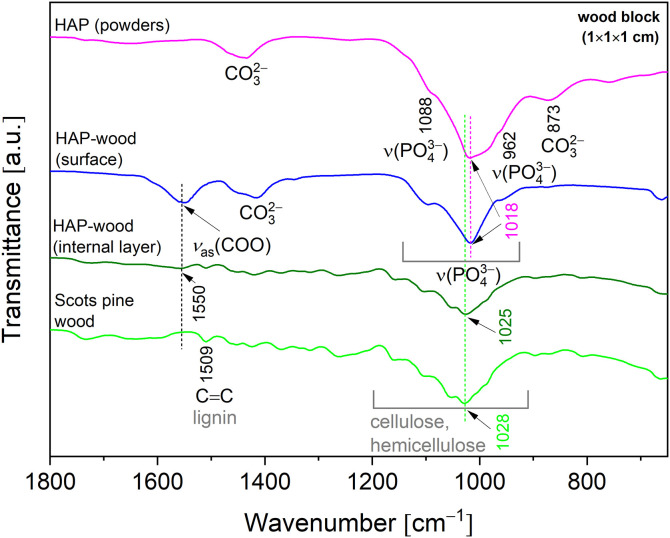
FTIR spectra of Scots pine wood, cHAP-mineralised wood, and co-precipitated cHAP powders.

The chemical composition of mineralised wood was further evaluated by FTIR spectroscopy by analysing the observed vibrational frequencies of molecular bonds, which are directly related to molecular functional groups and, subsequently, to constituents, *i.e.,* wood biomolecules and inorganic minerals. Fig 6 presents IR spectra of untreated Scots pine wood, mineralised wood surface and internal layers of the wood block, and CaP powders that co-precipitated from reactive species in an impregnation solution. The spectral region of 1800–650 cm^–1^ was selected to demonstrate the representative functional groups in these materials (the full spectra are presented in S3 Fig). Untreated wood showed typical IR spectral bands assignable to the main chemical components of wood, *i.e.*, cellulose, hemicellulose, and lignin [57,64]. HAP powders that co-precipitated within the parent solution showed intensive bands characteristic of the apatite mineral. The complex, broad band present in the 1100–950 cm^–1^ region comes from a triply degenerate asymmetric stretching mode, ν_3_, and a symmetric stretching mode, ν_1_, of the P–O bonds of the apatitic $\overset{\text{3}-}{\underset{\text{4}}{\text{PO}}}$ groups. The characteristic bands for the carbonate ($\overset{\text{2}-}{\underset{\text{3}}{\text{CO}}}$) group occur in the spectral regions 1600–1400 cm^–1^ for ν_3_ asymmetric stretch vibration, and 880–873 cm^–1^ for ν_2_ out-of-plane bend vibration, indicating the carbonate substitution within the HAP crystal lattice. The lack of sharply resolved bands ascribed to the carbonate group suggests an overlap between specific vibrations of this group, characteristic of A- and B-substituted carbonated hydroxyapatite (cHAP) with chemical formula defined as Ca_10-x_(PO_4_)_6-x_(CO_3_)_x_(OH)_2-x-2y_(CO_3_)_y_ [20,65]. Furthermore, these bands might be ascribed to the presence of CaCO_3_ phase [66]. IR spectral data from the surface of the mineralised wood block showed similar features to those observed for the cHAP powders. However, one distinctive spectral feature is a broad band in the 1580–1540 cm^–1^ region. This could be ascribed to the carboxylate (COO^–^) group present in acetate ions. In the case of the sample taken from the internal part of the mineralised wood block, the IR spectrum exhibited marginal changes compared to untreated wood. Cellulose gives vibrations from 900 to 1100 cm^–1^, with maximum at 1028 cm^–1^, and there were no noticeable changes in band intensities obtained after treatment. This indicates that a very marginal amount of cHAP mineral is present within the internal layers of the wood block, agreeing with the SEM/EDS data that a small amount of CaP mineral was formed within cell lumina.

### 3.4 Fire properties of mineralised wood

**Fig 7:**
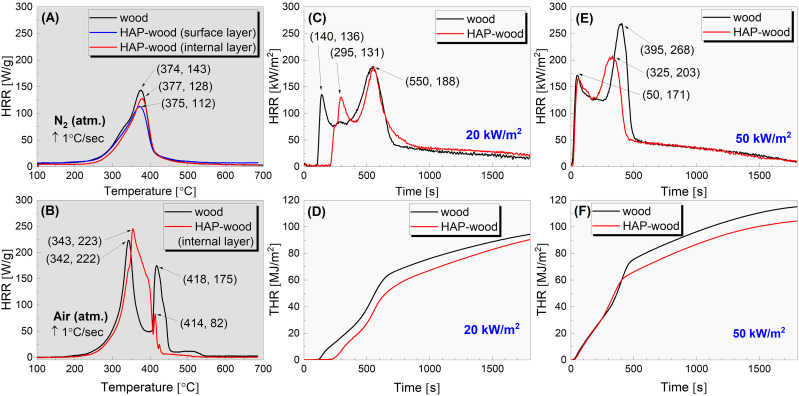
Microscale combustion calorimeter HRR curves of the cHAP-mineralised wood. (A) tests performed in N_2_ atmosphere and (B) in a synthetic air atmosphere (mass of samples was around 5 mg); and example test data from cone calorimeter: HRR and THR curves at (C, D) 20 kW/m^2^ and (E, F) 50 kW/m^2^ for cHAP-mineralised and untreated Scots pine wood.

Combustion of the HAP-mineralised wood was estimated from microscale samples using MCC, as well as larger samples via CC. MCC tests were performed in N_2_ and synthetic air atmospheres. The primary use of MCC is as a flammability assessment-screening tool for new materials [40]. Studying 100 μm stubs allowed for estimation of the mineral content within the entire wood block. MCC tests performed in N_2_ atmosphere (Fig 7A) showed a decrease in the peak heat release rate (HRR) per gram for the tested sample of mineralised wood. Specifically, the surface-layer sample showed about 22% lower peak HRR, whereas the internal-layer sample showed about 11% lower peak HRR. This data also showed a difference between the surface and internal layers of the mineralised wood block, suggesting that a mineral concentration gradient exists within the entire wood block and that impregnation is not consistent throughout the sample cross section. The HRR curves for MCC of Scots pine wood in a synthetic air atmosphere (Fig 7B) bear similarity to the results obtained from thermogravimetric analysis (TGA) tests reported elsewhere [36]. Different MCC results were obtained for the eggshell-cHAP-mineralised wood, which can be observed in the different shape of the HRR curve, in which the two reactions seen in the wood sample appear to be joined together. This might be caused by induced changes in the wood material due to treatment or by removal of extractives from the wood matrix due to the treatment solution’s alkalinity (pH 10–11). On the other hand, the coprecipitated low-crystallinity cHAP mineral and residue salts might act as a catalyst of the second reaction, represented by the second peak in the HRR curve of the untreated Scots pine, resulting in continuous heat release. Furthermore, the residue char left after burn-off was negligible for unmodified wood, whilst the eggshell-cHAP wood ash content was 4.9%, indicating that the mineral–wood composite possesses a higher density than untreated wood.

The CC test, performed at 20 kW/m^2^ and 50 kW/m^2^, provided further insight into the fire behaviour by presenting various important parameters, including HRR, THR, mass loss, and time to ignition (TTI), among others. CC data obtained for the untreated Scots pine wood and cHAP-mineralised wood are presented in Table 1. CC HRR and THR curves (Fig 7C–7F) show that the greatest impact of the mineral on the wood material was a delay in ignition time, seen in the tests performed under an external heat flux of 20 kW/m^2^. TTI is an essential indicator of fire initiation and fire behaviour. The HRR curves have similar shapes; however, ignition is delayed in the mineralised sample, indicating that the mineral may work as a heat sink. A similar behaviour was observed for aluminium hydroxide (Al(OH)_3_) [67]. Note also that the degradation temperature of mineralised wood is slightly shifted to lower temperatures [34,57]. The second peak in the HRR curves is related to reduced heat losses when the thermal penetration reaches the rear of the sample. This is due to the insulation layer (ceramic wool) underneath the sample in the sample holder, which leads to a higher temperature at the back side of the sample, inducing an increased burning rate, and thus a higher HRR [68]. Moreover, the mineralised wood samples exhibited similar behaviour during CC and MCC tests, i.e., under heat flux of 50 kW/m^2^, the second peak occurred sooner than for the untreated wood. Furthermore, THR was slightly reduced for mineralised wood for both heat fluxes (Fig 7D, 7F). To confirm the course of this event, further in-depth studies shall be undertaken. Further investigations are also needed to determine how the initial solution concentration, the wood treatment itself, and the wood drying steps affect the homogeneity of mineral distribution within the wood matrix.

**Table 1:** CC data for untreated Scots pine wood and cHAP-wood specimens (± values represent SD).

Properties	Heat flux (kW m − 2)	Reference: untreated wood	Sample: cHAP-wood
Time to ignition (s)	20	120 ± 7	250 ± 35
	50	23 ± 3	28 ± 3
Critical heat flux (kW m ^− 2^) calculated in compliance with Spearpoint [69]	NA	17	22
Ignition temperature (°C) calculated in compliance with Spearpoint [69]	NA	388	442
Time to flashover (s) calculated in compliance with Kokkala [39]	NA	120–600	120–600
Maximum heat release rate (kW m ^− 2^)	20	197 ± 3	199 ± 33
	50	273 ± 13	252 ± 26
Maximum average rate of heat emission (kW m ^− 2^)	20	97 ± 4	80 ± 6
	50	157 ± 2	152 ± 2
Total heat release (MJ m ^− 2^)	20	94 ± 11	91 ± 2
	50	115 ± 4	104 ± 12
Effective heat of combustion (MJ kg ^− 1^)	20	16.22 ± 0.77	15.29 ± 0.70
	50	17.85 ± 0.28	16.92 ± 0.05
Maximum mass loss rate (g m ^− 2^ s ^− 1^)	20	16.45 ± 1.70	21.08 ± 2.50
	50	20.13 ± 0.21	21.36 ± 0.57
Average mass loss rate (g m ^− 2^ s ^− 1^)	20	3.22 ± 0.23	3.29 ± 0.21
	50	3.58 ± 0.07	3.42 ± 0.39

The work on eggshell processing to extract proteins and mineralise wood to improve wood-reaction-to-fire attested the importance of material recycling. The composition of eggshell biowaste offers a pool of valuable products with many applications. Demonstrated closed-loop strategy with food processing waste disposal being reduced as much as possible could provide a pathway for the development of new green technologies using eggshells as a raw material for novel bio-based materials. A strategy for future development in biowaste management is essential and should involve economic, environmental, and social aspects aiming to accomplish sustainable development.

## 4 Conclusions

We have investigated the processing of biowaste eggshells via a wet-chemistry approach and demonstrated the pathway to utilise both the organic and inorganic components of the eggshell material. First, water-insoluble proteins were successfully extracted by boiling in water with a dissociating/reducing buffer. The proteins ovocalyxin, ovocleidin and lysozyme were identified in the eggshell matrix, while ovoglycoprotein, ovocalyxin and ovotransferrin were identified in the eggshell membranes. Additionally, the eggshells were processed to prepare aqueous Ca(CH_3_COO)_2_ solution, which was used together with an aqueous NH_4_H_2_PO_4_ solution to mineralise the wood matrix via a two-step impregnation process at 20–23 °C. Low wood matrix saturation, *i.e.,* unfilled cell lumina with mineral, with homogeneous Ca and P distribution was observed. The *in situ* coprecipitated mineral was shown to possess a low-crystallinity cHAP phase. The formation of the HAP phase is apparently related to the precursor solution pH of 10–11. Carbonated HAP formation is implied by the IR spectroscopy data, as the spectra of mineralised wood exhibit strong bands characteristic of apatitic $\overset{\text{3}-}{\underset{\text{4}}{\text{PO}}}$ and $\overset{\text{2}-}{\underset{\text{3}}{\text{CO}}}$ groups. Mineralisation of wood resulted in a reduction in total heat release, indicating that the mineral may work as a heat sink for bio-based materials and suggesting potential for further optimisation of wood modification process. The results suggest that the proposed aqueous solution-based processing approach for converting an abundant resource, chicken eggshells, into value-added products has potential for new technology and bioeconomy development and represents a promising pathway towards improved sustainability.

## Supporting information

S1 Fig Original post-electrophoresis gel of the eggshell matrix. (PDF)

S2 Fig Original SDS-PAGE gel of the eggshell matrix showing the marked well positions of samples with loading volumes of 10 µL and 20 µL. (PDF)

S3 Fig FTIR spectra of Scots pine wood, cHAP-mineralised wood (internal and surface layers), and co-precipitated cHAP powders (shown full recorded region of 4000 cm^–1^–650 cm^–1^). (PDF)

## References

[1] Kircher M, Aranda E, Athanasios P, Radojcic-Rednovnikov I, Romantschuk M, Ryberg M, et al. Treatment and valorization of bio-waste in the EU. EFB Bioeconomy Journal. 2023;3:100051. doi: 10.1016/j.bioeco.2023.100051

[2] Seadi TA, Holm-Nielsen JBI. Agricultural wastes. In: Twardowska I, editor. Waste Management Series. Elsevier; 2004. p. 207–15. doi: 10.1016/S0713-2743(04)80011-4

[3] Gardossi L, Philp J, Fava F, Winickoff D, D’Aprile L, Dell’Anno B, et al. Bioeconomy national strategies in the G20 and OECD countries: Sharing experiences and comparing existing policies. EFB Bioeconomy Journal. 2023;3:100053. doi: 10.1016/j.bioeco.2023.100053

[4] Quina MJ, Soares MAR, Quinta-Ferreira R. Applications of industrial eggshell as a valuable anthropogenic resource. Resour Conserv Recycl. 2017;123:176–86. doi: 10.1016/j.resconrec.2016.09.027

[5] Soares MAR, Quina MMJ, Quinta-Ferreira RM. Co-composting of eggshell waste in self-heating reactors: monitoring and end product quality. Bioresour Technol. 2013;148:293–301. doi: 10.1016/j.biortech.2013.08.151 24055972

[6] FMI Report. Egg and Egg Products Market (2026 - 2036). Future Market Insights. 2026. https://www.futuremarketinsights.com/reports/egg-products-market

[7] Waheed M, Yousaf M, Shehzad A, Inam-Ur-Raheem M, Khan MKI, Khan MR, et al. Channelling eggshell waste to valuable and utilizable products: a comprehensive review. Trends in Food Science & Technology. 2020;106:78–90. doi: 10.1016/j.tifs.2020.10.009

[8] Ngayakamo B, Onwualu AP. Recent advances in green processing technologies for valorisation of eggshell waste for sustainable construction materials. Heliyon. 2022;8(6):e09649. doi: 10.1016/j.heliyon.2022.e09649 35711973 PMC9193877

[9] Vandeginste V. Food waste eggshell valorization through development of new composites: a review. Sustain Mater Technol. 2021;29:e00317. doi: 10.1016/j.susmat.2021.e00317

[10] Hincke MT, Nys Y, Gautron J, Mann K, Rodriguez-Navarro AB, McKee MD. The eggshell: structure, composition and mineralization. Front Biosci (Landmark Ed). 2012;17(4):1266–80. doi: 10.2741/3985 22201802

[11] Gautron J, Stapane L, Le Roy N, Nys Y, Rodriguez-Navarro AB, Hincke MT. Avian eggshell biomineralization: an update on its structure, mineralogy and protein tool kit. BMC Mol Cell Biol. 2021;22(1):11. doi: 10.1186/s12860-021-00350-0 33579194 PMC7881572

[12] Rose MLH, Hincke MT. Protein constituents of the eggshell: eggshell-specific matrix proteins. Cell Mol Life Sci. 2009;66(16):2707–19. doi: 10.1007/s00018-009-0046-y 19452125 PMC11115492

[13] Han C, Chen Y, Shi L, Chen H, Li L, Ning Z, et al. Advances in eggshell membrane separation and solubilization technologies. Front Vet Sci. 2023;10:1116126. doi: 10.3389/fvets.2023.1116126 37008347 PMC10060898

[14] Ahmed TAE, Suso H-P, Hincke MT. In-depth comparative analysis of the chicken eggshell membrane proteome. J Proteomics. 2017;155:49–62. doi: 10.1016/j.jprot.2017.01.002 28087451

[15] Cordeiro CMM, Hincke MT. Recent patents on eggshell: shell and membrane applications. Recent Pat Food Nutr Agric. 2011;3(1):1–8. doi: 10.2174/2212798411103010001 21114472

[16] Ahmed TAE, Wu L, Younes M, Hincke M. Biotechnological applications of eggshell: recent advances. Front Bioeng Biotechnol. 2021;9:675364. doi: 10.3389/fbioe.2021.675364 34295881 PMC8291997

[17] Richter R, Lee EJ, Grant JE. Optimization of Extraction and Isolation of Proteins from Eggshells. The FASEB Journal. 2018;32(S1). doi: 10.1096/fasebj.2018.32.1_supplement.530.32

[18] Le Roy N, Stapane L, Gautron J, Hincke MT. Evolution of the avian eggshell biomineralization protein toolkit - new insights from multi-omics. Front Genet. 2021;12:672433. doi: 10.3389/fgene.2021.672433 34046059 PMC8144736

[19] LeGeros RZ, Lin S, Rohanizadeh R, Mijares D, LeGeros JP. Biphasic calcium phosphate bioceramics: preparation, properties and applications. J Mater Sci Mater Med. 2003;14(3):201–9. doi: 10.1023/a:1022872421333 15348465

[20] Garskaite E, Gross KA, Yang SW, Yang TCK, Yang JC, Kareiva A. Effect of processing conditions on the crystallinity and structure of carbonated calcium hydroxyapatite (CHAp). CrystEngComm. 2014;16(19):3950–9. doi: 10.1039/C4CE00119B

[21] Grigoraviciute-Puroniene I, Tanaka Y, Vegelyte V, Nishimoto Y, Ishikawa K, Kareiva A. A novel synthetic approach to low-crystallinity calcium deficient hydroxyapatite. Ceram Int. 2019;45(12):15620–3. doi: 10.1016/j.ceramint.2019.05.072

[22] Dodoo A, Gustavsson L, Sathre R. Lifecycle primary energy analysis of low-energy timber building systems for multi-storey residential buildings. Energy and Buildings. 2014;81:84–97. doi: 10.1016/j.enbuild.2014.06.003

[23] Lowden L, Hull T. Flammability behaviour of wood and a review of the methods for its reduction. Fire Sci Rev. 2013;2(1):4. doi: 10.1186/2193-0414-2-4

[24] Popescu C, Pfriem A. Treatments and modification to improve the reaction to fire of wood and wood based products—an overview. Fire and Materials. 2019;44(1):100–11. doi: 10.1002/fam.2779

[25] Jones D, Lin CF, Kim I, Garskaite E, Karlsson O, Sandberg D. Recent studies into improved fire retardancy of wood undertaken at Luleå University of Technology. In: Proceedings IRG54 Scientific Conference on Wood Protection. Cairns, Australia. 2023.

[26] Ehrhardt C, Tapken M, Namyslo JC, Kaufmann DE. Chemical improvement of surfaces. Part 6: Enhanced flame retardancy of Scots pine sapwood by covalent modification with phosphorus and boron functionalized benzoates. Holzforschung. 2021;75(1):83–90. doi: 10.1515/hf-2020-0041

[27] Kong L, Guan H, Wang X. In situ polymerization of furfuryl alcohol with ammonium dihydrogen phosphate in poplar wood for improved dimensional stability and flame retardancy. ACS Sustainable Chem Eng. 2018;6(3):3349–57. doi: 10.1021/acssuschemeng.7b03518

[28] Lin C-F, Karlsson O, Martinka J, Rantuch P, Garskaite E, Mantanis GI, et al. Approaching highly leaching-resistant fire-retardant wood by in situ polymerization with melamine formaldehyde resin. ACS Omega. 2021;6(19):12733–45. doi: 10.1021/acsomega.1c01044 34056425 PMC8154219

[29] Sauerbier P, Mayer AK, Emmerich L, Militz H. Fire retardant treatment of wood – state of the art and future perspectives. Wood & Fire Safety. Cham: Springer; 2020. p. 97–102.

[30] Saka S, Ueno T. Several SiO2 wood-inorganic composites and their fire-resisting properties. Wood SciTechnol. 1997;31(6):457–66. doi: 10.1007/bf00702568

[31] Merk V, Chanana M, Keplinger T, Gaan S, Burgert I. Hybrid wood materials with improved fire retardance by bio-inspired mineralisation on the nano- and submicron level. Green Chem. 2015;17(3):1423–8. doi: 10.1039/c4gc01862a

[32] Pondelak A, Škapin AS, Knez N, Knez F, Pazlar T. Improving the flame retardancy of wood using an eco-friendly mineralisation process. Green Chem. 2021;23(3):1130–5. doi: 10.1039/d0gc03852k

[33] Guo H, Luković M, Mendoza M, Schlepütz CM, Griffa M, Xu B, et al. Bioinspired struvite mineralization for fire-resistant wood. ACS Appl Mater Interfaces. 2019;11(5):5427–34. doi: 10.1021/acsami.8b19967 30623641

[34] Garskaite E, Balciunas G, Drienovsky M, Sokol D, Sandberg D, Bastos AC, et al. Brushite mineralised Scots pine (Pinus sylvestris L.) sapwood - revealing mineral crystallization within a wood matrix by in situ XRD. RSC Adv. 2023;13(9):5813–25. doi: 10.1039/d3ra00305a 36816063 PMC9932638

[35] Bohner M, Santoni BLG, Döbelin N. β-tricalcium phosphate for bone substitution: Synthesis and properties. Acta Biomater. 2020;113:23–41. doi: 10.1016/j.actbio.2020.06.022 32565369

[36] Garskaite E. In-situ synthesis of calcium phosphates derived from eggshells to improve wood reaction-to-fire properties. 2021–013. 2022.

[37] ISO 5660-1:2015. Reaction to fire tests. Heat release, smoke production and mass loss rate. Part 1: Heat release rate (cone calorimeter method) and smoke production rate (dynamic measurement). International Organization for Standardization. 2015. https://www.iso.org/standard/66683.html

[38] Marquis D, Guillaume E, Lesenechal D. Accuracy (trueness and precision) of cone calorimeter tests with and without a vitiated air enclosure. Procedia Engineering. 2013;62:103–19. doi: 10.1016/j.proeng.2013.08.048

[39] Kokkala MA, Thomas PH, Karlsson B. Rate of heat release and ignitability indices for surface linings. Fire and Materials. 1993;17(5):209–16. doi: 10.1002/fam.810170503

[40] Wilkens Flecknoe-Brown K. Fire behaviour of upholstered furniture component materials at multiple scales. Sweden: Lund University; 2022.

[41] Mine Y, Oberle C, Kassaify Z. Eggshell matrix proteins as defense mechanism of avian eggs. J Agric Food Chem. 2003;51(1):249–53. doi: 10.1021/jf020597x 12502416

[42] Hincke MT, Gautron J, Tsang CP, McKee MD, Nys Y. Molecular cloning and ultrastructural localization of the core protein of an eggshell matrix proteoglycan, ovocleidin-116. J Biol Chem. 1999;274(46):32915–23. doi: 10.1074/jbc.274.46.32915 10551857

[43] Miksík I, Eckhardt A, Sedláková P, Mikulikova K. Proteins of insoluble matrix of avian (gallus gallus) eggshell. Connect Tissue Res. 2007;48(1):1–8. doi: 10.1080/03008200601003116 17364661

[44] Gautron J, Réhault-Godbert S, Pascal G, Nys Y, Hincke MT. Ovocalyxin-36 and other LBP/BPI/PLUNC-like proteins as molecular actors of the mechanisms of the avian egg natural defences. Biochem Soc Trans. 2011;39(4):971–6. doi: 10.1042/BST0390971 21787332

[45] Hernández-Hernández A, Gómez-Morales J, Rodríguez-Navarro AB, Gautron J, Nys Y, García-Ruiz JM. Identification of some active proteins in the process of hen eggshell formation. Crystal Growth & Design. 2008;8(12):4330–9. doi: 10.1021/cg800786s

[46] Hincke MT, Gautron J, Panheleux M, Garcia-Ruiz J, McKee MD, Nys Y. Identification and localization of lysozyme as a component of eggshell membranes and eggshell matrix. Matrix Biol. 2000;19(5):443–53. doi: 10.1016/s0945-053x(00)00095-0 10980420

[47] Kaweewong K, Garnjanagoonchorn W, Jirapakkul W, Roytrakul S. Solubilization and identification of hen eggshell membrane proteins during different times of chicken embryo development using the proteomic approach. Protein J. 2013;32(4):297–308. doi: 10.1007/s10930-013-9487-0 23636516

[48] Panheleux M, Nys Y, Williams J, Gautron J, Boldicke T, Hincke MT. Extraction and quantification by ELISA of eggshell organic matrix proteins (ovocleidin-17, ovalbumin, ovotransferrin) in shell from young and old hens. Poult Sci. 2000;79(4):580–8. doi: 10.1093/ps/79.4.580 10780658

[49] Tadeo X, López-Méndez B, Castaño D, Trigueros T, Millet O. Protein stabilization and the Hofmeister effect: the role of hydrophobic solvation. Biophys J. 2009;97(9):2595–603. doi: 10.1016/j.bpj.2009.08.029 19883603 PMC2770621

[50] He X, Ewing AG. Hofmeister series: from aqueous solution of biomolecules to single cells and nanovesicles. Chembiochem. 2023;24(9):e202200694. doi: 10.1002/cbic.202200694 37043703

[51] Demir İ, Karakaya N, Akdemir Evrendilek G, Turan S. Valorization of egg shell membrane as protein source in soft gel capsules. Waste Biomass Valor. 2024;15(8):5025–41. doi: 10.1007/s12649-024-02519-y

[52] Ahlborn GJ, Clare DA, Sheldon BW, Kelly RW. Identification of eggshell membrane proteins and purification of ovotransferrin and beta-NAGase from hen egg white. Protein J. 2006;25(1):71–81. doi: 10.1007/s10930-006-0010-8 16721662

[53] Jonchère V, Réhault-Godbert S, Hennequet-Antier C, Cabau C, Sibut V, Cogburn LA, et al. Gene expression profiling to identify eggshell proteins involved in physical defense of the chicken egg. BMC Genomics. 2010;11:57. doi: 10.1186/1471-2164-11-57 20092629 PMC2827412

[54] Cordeiro CMM, Esmaili H, Ansah G, Hincke MT. Ovocalyxin-36 is a pattern recognition protein in chicken eggshell membranes. PLoS One. 2013;8(12):e84112. doi: 10.1371/journal.pone.0084112 24391897 PMC3877205

[55] Makkar S, Liyanage R, Kannan L, Packialakshmi B, Lay Jr JO, Rath NC. Chicken egg shell membrane associated proteins and peptides. J Agric Food Chem. 2015;63(44):9888–98. doi: 10.1021/acs.jafc.5b04266 26485361

[56] Cree D, Rutter A. Sustainable bio-inspired limestone eggshell powder for potential industrialized applications. ACS Sustainable Chem Eng. 2015;3(5):941–9. doi: 10.1021/acssuschemeng.5b00035

[57] Garskaite E, Karlsson O, Stankeviciute Z, Kareiva A, Jones D, Sandberg D. Surface hardness and flammability of Na2SiO3 and nano-TiO2 reinforced wood composites. RSC Advances. 2019;9(48):27973–86. doi: 10.1039/C9RA05200C 35530478 PMC9071003

[58] Wang Y, Tian T, Cabane E. Wood composites with wettability patterns prepared by controlled and selective chemical modification of a three-dimensional wood scaffold. ACS Sustainable Chem Eng. 2017;5(12):11686–94. doi: 10.1021/acssuschemeng.7b03104

[59] Burgert I, Cabane E, Zollfrank C, Berglund L. Bio-inspired functional wood-based materials – hybrids and replicates. Int Mater Rev. 2015;60(8):431–50. doi: 10.1179/1743280415Y.0000000009

[60] Garskaite E, Alinauskas L, Drienovsky M, Krajcovic J, Cicka R, Palcut M, et al. Fabrication of a composite of nanocrystalline carbonated hydroxyapatite (cHAP) with polylactic acid (PLA) and its surface topographical structuring with direct laser writing (DLW). RSC Adv. 2016;6(76):72733–43. doi: 10.1039/c6ra11679e

[61] Kapolos J, Koutsoukos PG. Formation of calcium phosphates in aqueous solutions in the presence of carbonate ions. Langmuir. 1999;15(19):6557–62. doi: 10.1021/la981285k

[62] Upadhyay P, Ullah A. Facile synthesis of hydroxyapatite nanoparticles from eggshell biowaste usingAzadirachta indicaextract as a green template. New J Chem. 2024;48(3):1424–35. doi: 10.1039/d3nj01715j

[63] Garskaite E, Asuigui DR, Stoll SL, Hansson L, Sandberg D. Formation of glassy MgO/Na2O–SiO2 solid within Scots pine (Pinus sylvestris L.) sapwood through a wet-chemistry approach. New Journal of Chemistry. 2026;50:5687–99. doi: 10.1039/D6NJ00470A

[64] Garskaite E, Stoll SL, Forsberg F, Lycksam H, Stankeviciute Z, Kareiva A, et al. The accessibility of the cell wall in scots pine (Pinus sylvestris L.) sapwood to colloidal Fe3O4 nanoparticles. ACS Omega. 2021;6(33):21719–29. doi: 10.1021/acsomega.1c03204 34471774 PMC8388106

[65] Fleet ME. Infrared spectra of carbonate apatites: v2-Region bands. Biomaterials. 2009;30(8):1473–81. doi: 10.1016/j.biomaterials.2008.12.007 19111895

[66] Reig FB, Adelantado JVG, Moya Moreno MCM. FTIR quantitative analysis of calcium carbonate (calcite) and silica (quartz) mixtures using the constant ratio method. Application to geological samples. Talanta. 2002;58(4):811–21. doi: 10.1016/s0039-9140(02)00372-7 18968811

[67] Chai G, Zhu G, Gao S, Zhou J, Gao Y, Wang Y. On improving flame retardant and smoke suppression efficiency of epoxy resin doped with aluminum tri-hydroxide. Adv Compos Lett. 2019;28:2633366X19894597. doi: 10.1177/2633366X19894597

[68] Sanned E, Mensah RA, Försth M, Das O. The curious case of the second/end peak in the heat release rate of wood: a cone calorimeter investigation. Fire and Materials. 2022;47(4):498–513. doi: 10.1002/fam.3122

[69] Spearpoint MJ, Quintiere JG. Predicting the piloted ignition of wood in the cone calorimeter using an integral model—effect of species, grain orientation and heat flux. Fire Safety Journal. 2001;36(4):391–415. doi: 10.1016/s0379-7112(00)00055-2

