# Supplementary figures and images for: Circular economy approach to eggshell waste utilisation: Insoluble protein extraction and CaCO3 upcycling for carbonated hydroxyapatite (cHAP)-based fire-resistant wood
Source: PLoS One. 2026 Jun 25;21(6):e0351943. doi: 10.1371/journal.pone.0351943 (PMC13298751; doi:10.1371/journal.pone.0351943)

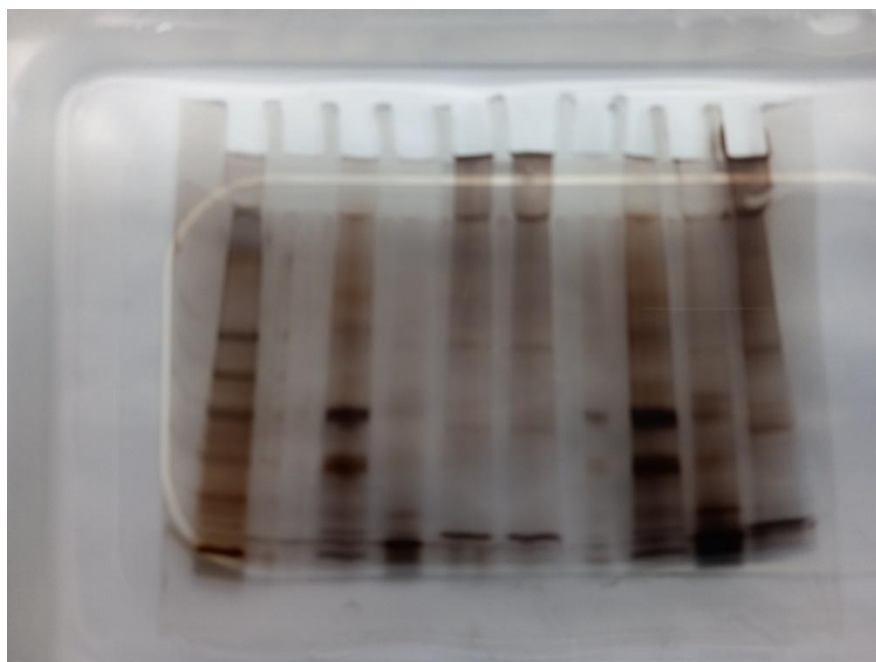

Supplement: S1 Fig — (PDF) [file pone.0351943.s001.pdf]

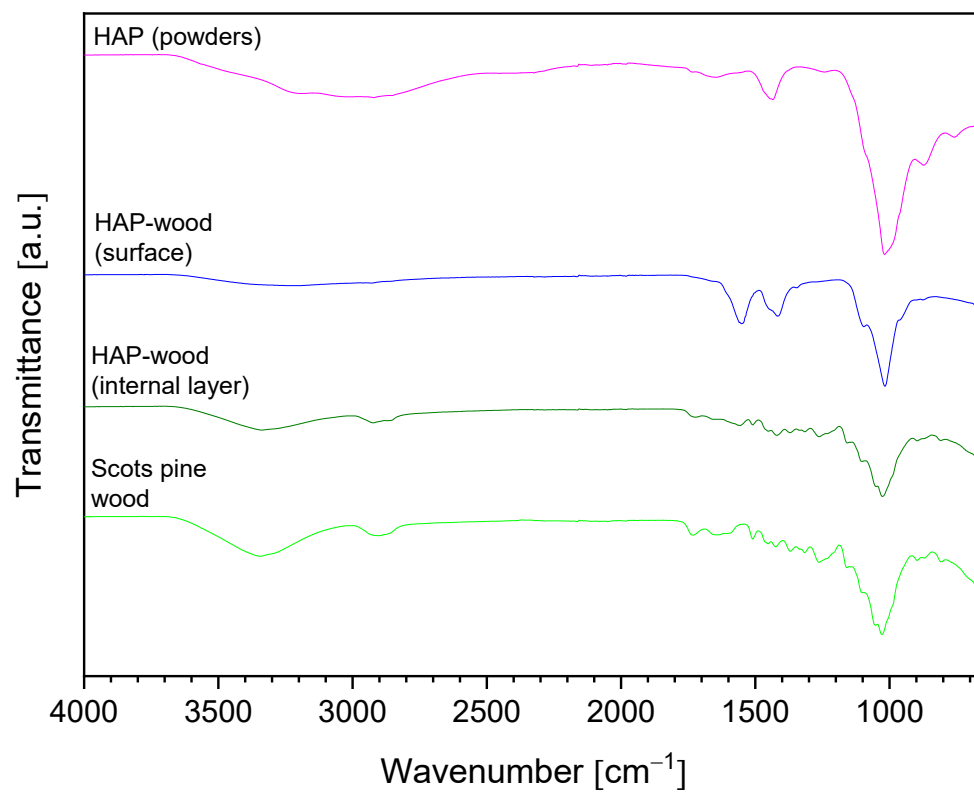

Supplement: S3 Fig — (PDF) [file pone.0351943.s003.pdf]
