# Supplementary material for: Circular economy approach to eggshell waste utilisation: Insoluble protein extraction and CaCO3 upcycling for carbonated hydroxyapatite (cHAP)-based fire-resistant wood
Source: PLoS One. 2026 Jun 25;21(6):e0351943. doi: 10.1371/journal.pone.0351943 (PMC13298751; doi:10.1371/journal.pone.0351943)
Supplement: S2 Fig — (PDF) [file pone.0351943.s002.pdf]

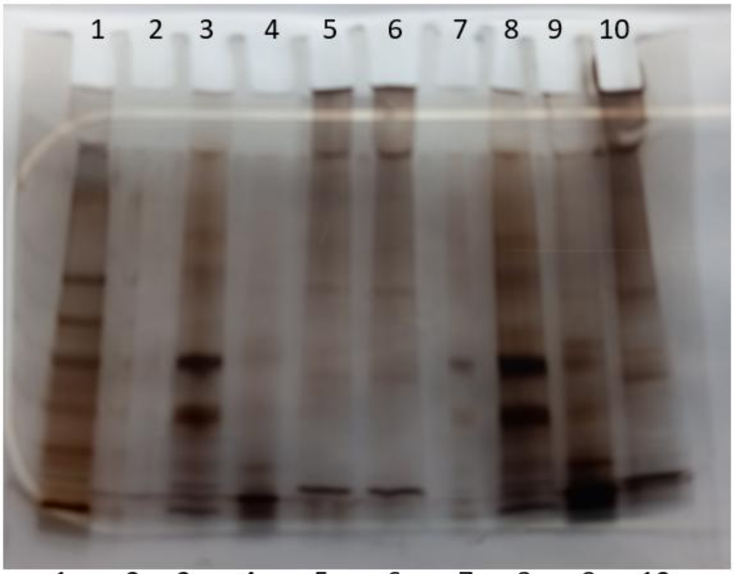

1 2 3 4 5 6 7 8 9 10

1 - reference ladder

2 - blank

3 - **10 µL** eggshell extract

4 - **10 µL** eggshell membrane

7 - blank

8 - **20 µL** eggshell extract

9 - **20 µL** eggshell membrane
